# Supplementary figures and images for: Dysfunction of Oskyddad causes Harlequin-type ichthyosis-like defects in Drosophila melanogaster
Source: PLoS Genet. 2020 Jan 13;16(1):e1008363. doi: 10.1371/journal.pgen.1008363 (PMC6980720; doi:10.1371/journal.pgen.1008363)

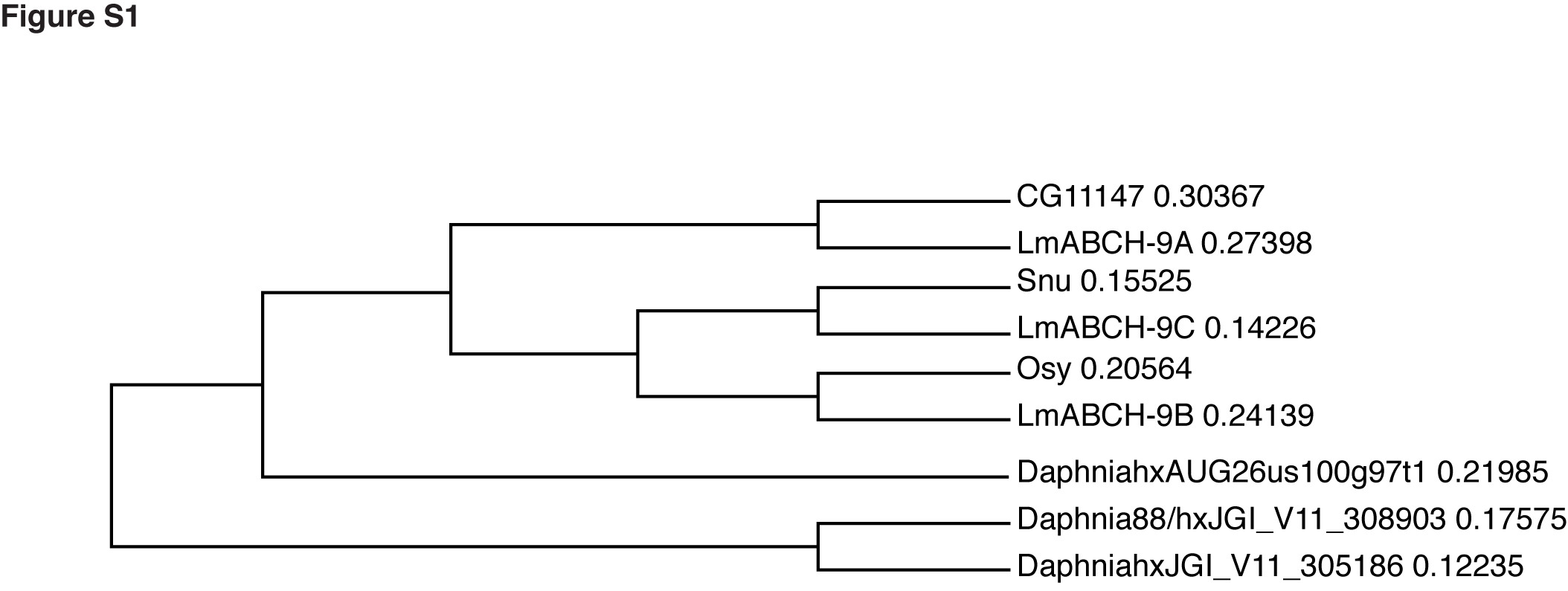

Supplement: S1 Fig — The ancestral protein that is shared with crustaceans (Daphnia), is ABCH-9A (CG11147 in D. melanogaster). (TIF) [file pgen.1008363.s001.tif]

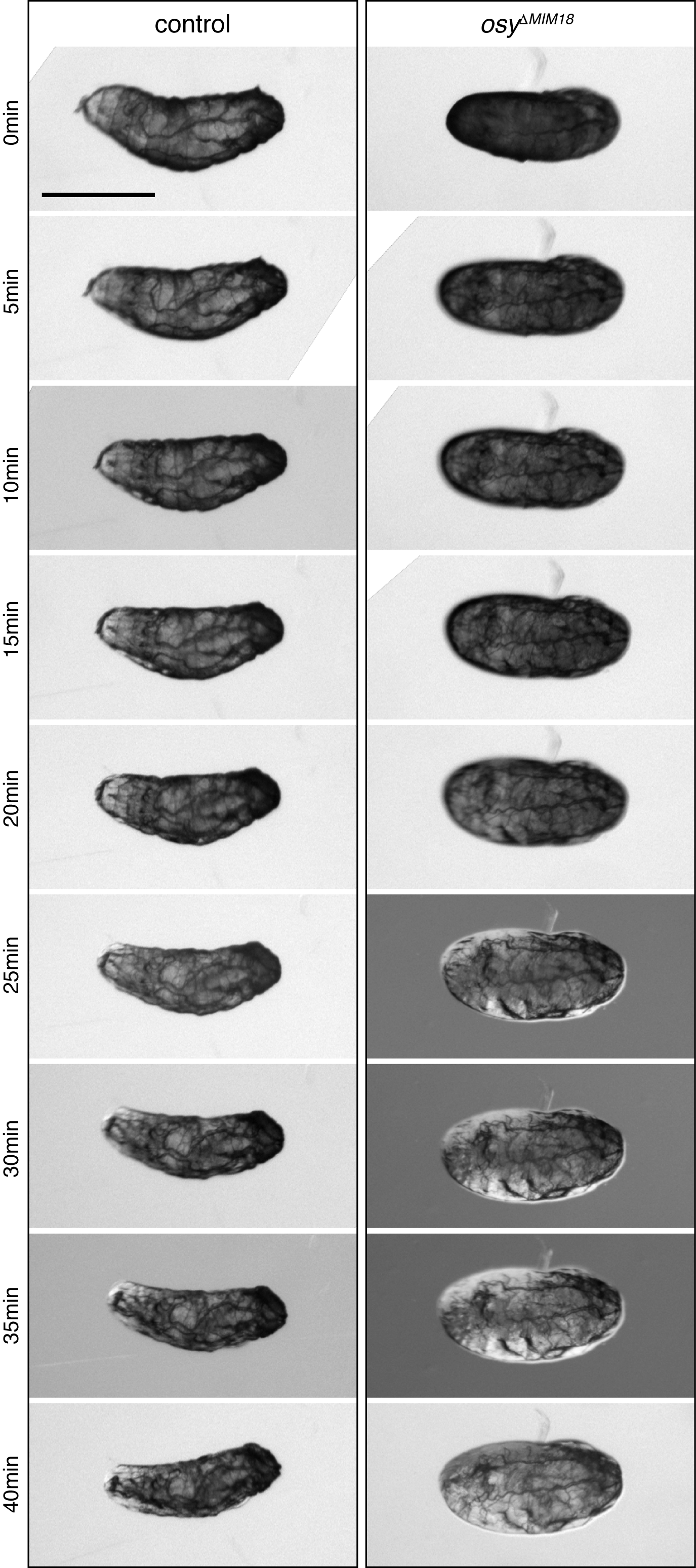

Supplement: S2 Fig — The progeny of this cross was viable to adulthood. Three independent experiments were conducted. For qPCR analyses, two technical replicates per experiment were performed. (TIF) [file pgen.1008363.s002.tif]

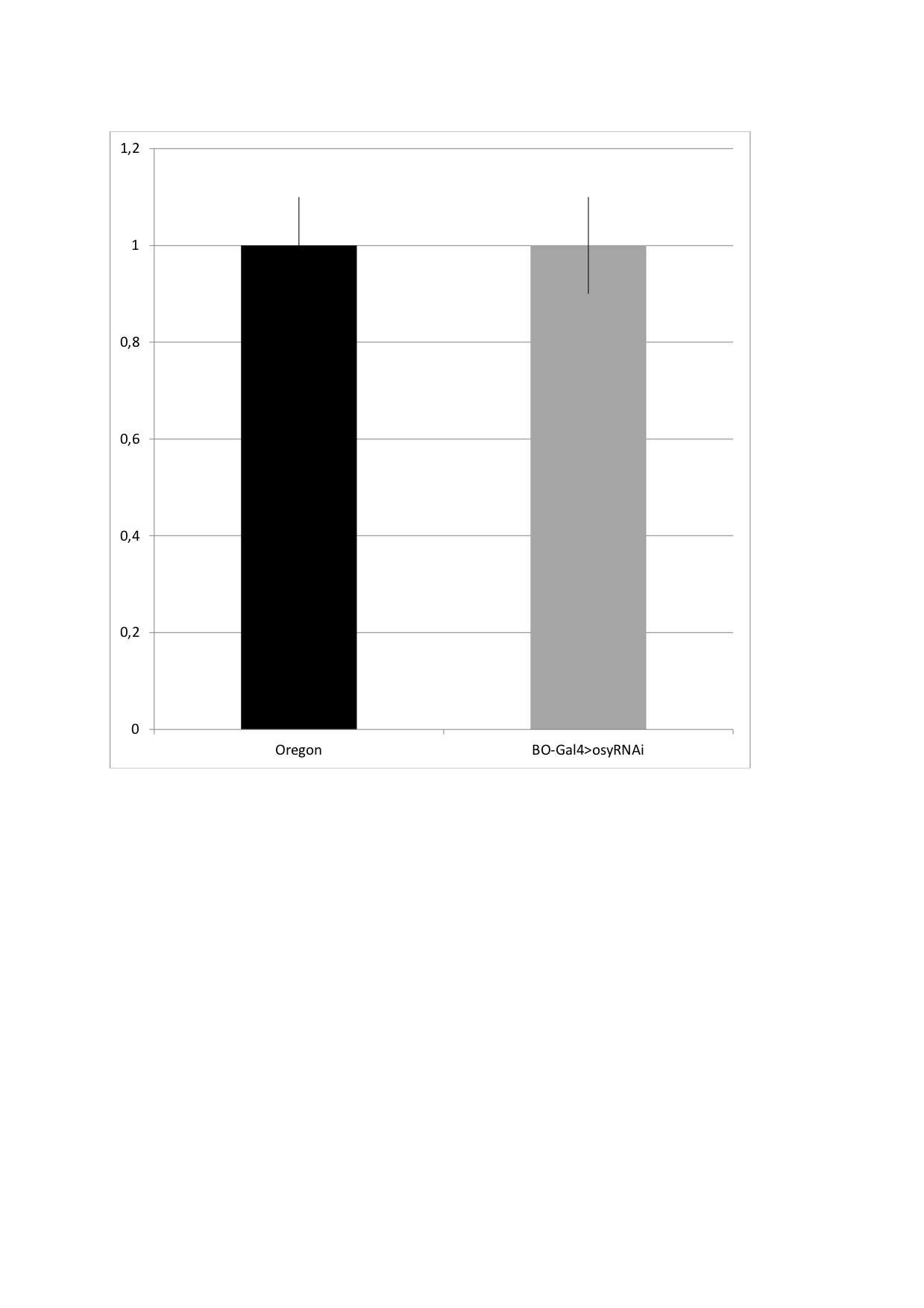

Supplement: S3 Fig — Often they are immobile before they start crawling to a food source. Larvae with eliminated osy function (osyΔMiM18) at around 15 minutes after hatching stop moving and start flattening. See also supplementary movies 1 and 2. (TIFF) [file pgen.1008363.s003.tiff]

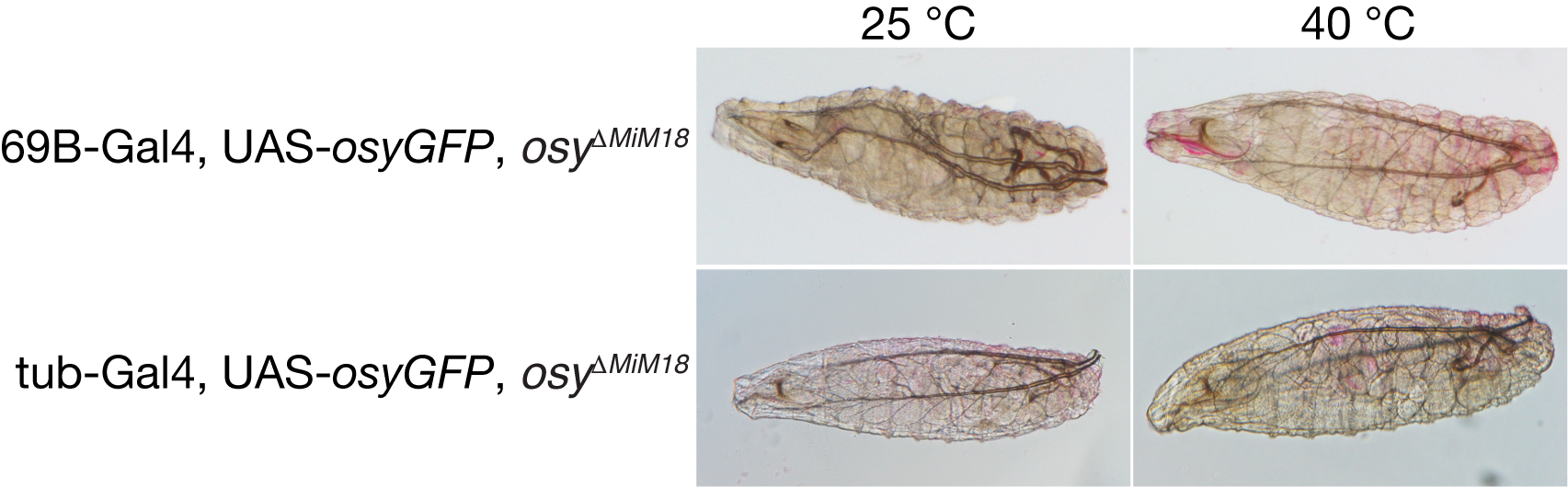

Supplement: S4 Fig — (TIF) [file pgen.1008363.s004.tif]

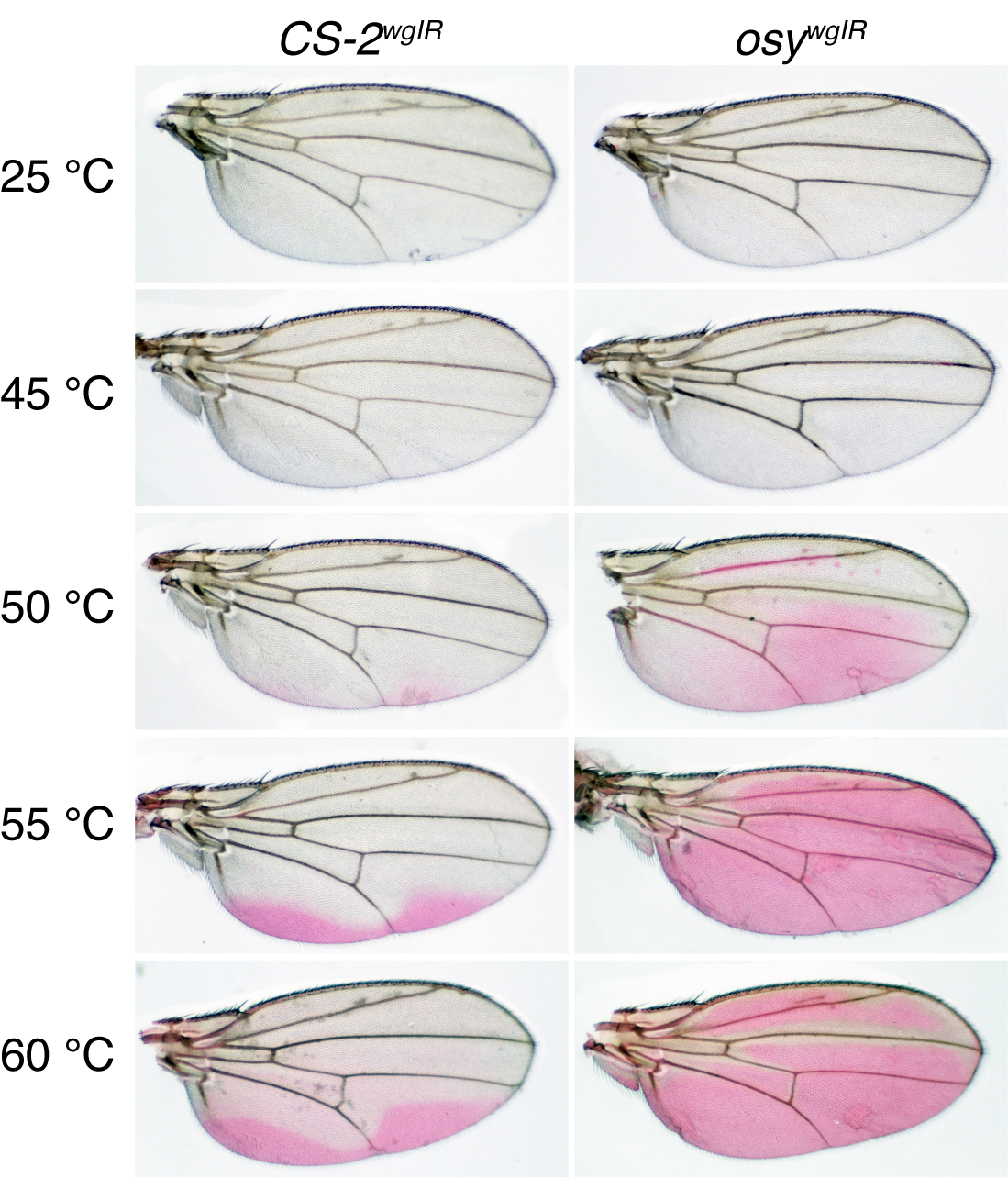

Supplement: S5 Fig — At 55°C, the dye penetrates the posterior margin of the wing of these flies. Penetration is more pronounced at higher temperatures (60°C). Wings of flies expressing hpRNA against osy (nub-Gal4 x UAS-KK109988, osywgIR) transcripts are impermeable to Eosin Y until 45°C. Eosin Y penetrates the posterior half of the wing of these flies at 50°C, and the whole wing at 55°C. It should be noted that osywgIR wings did not show any obvious morphological defect and the respective flies survived and did not desiccate. (TIF) [file pgen.1008363.s005.tif]

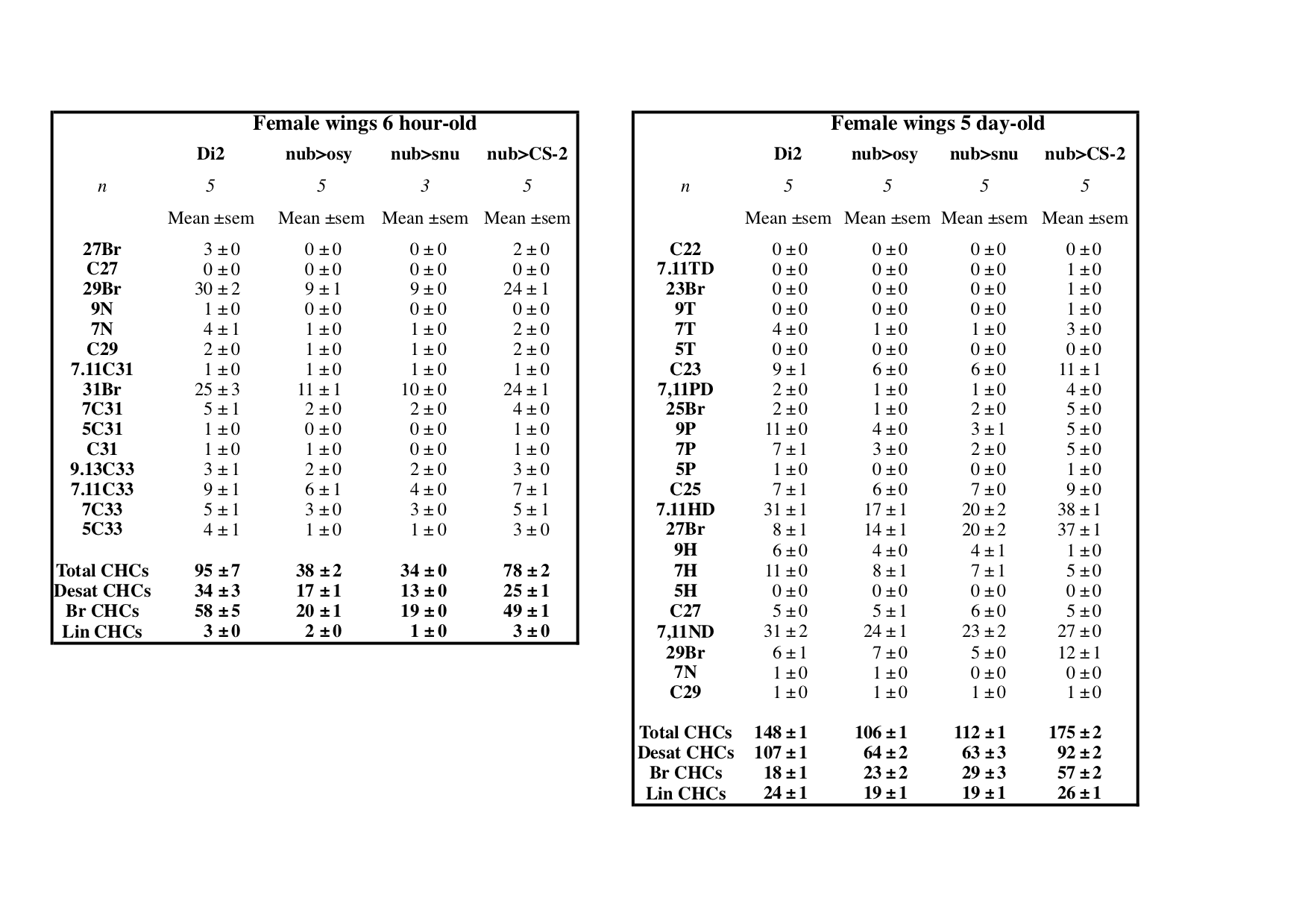

Supplement: S6 Fig — (TIFF) [file pgen.1008363.s006.tiff]

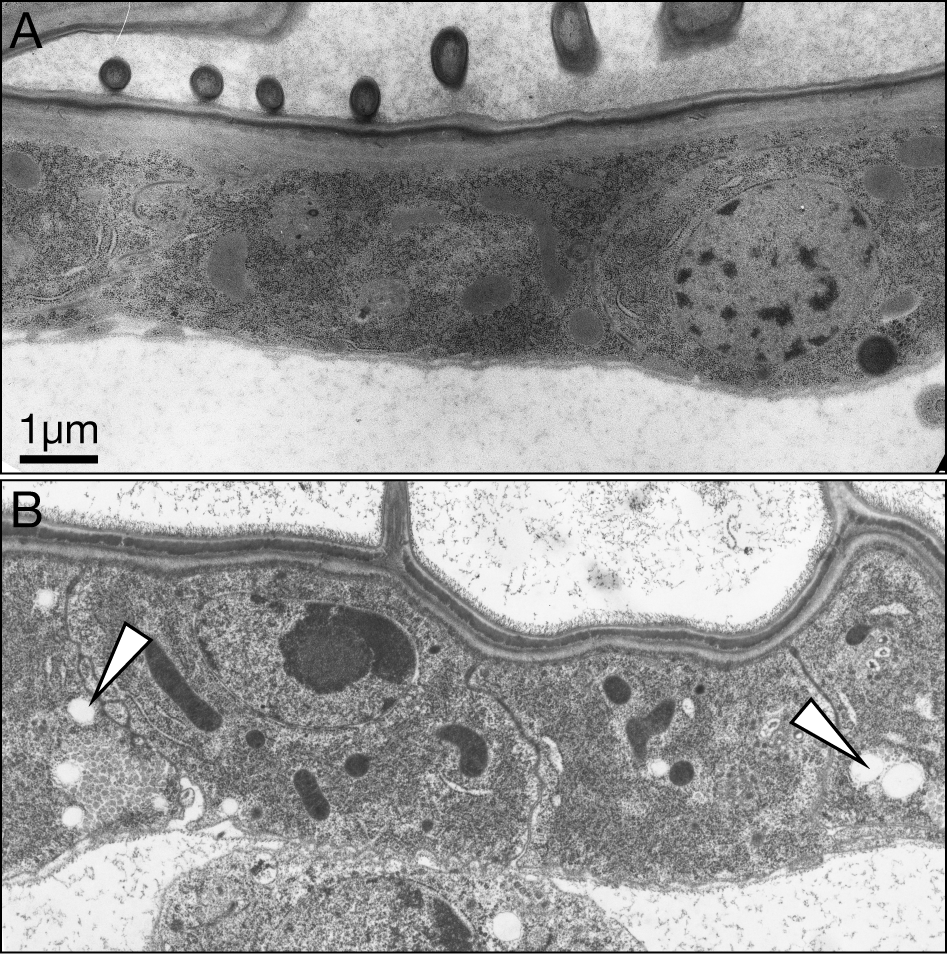

Supplement: S7 Fig — Electron-micrographs of the epidermis of wild-type (A) and osyΔMiM18 first instar larvae (B). Electron-lucid round structures (triangles) probably representing lipid droplet-like organelles were found in the osyΔMiM18 epidermal cells. These structures were missing in the wild-type control epidermis. Due to preparation of the specimens with acetone, lipids are usually extracted from the probes. Therefore, these structures are electron-lucid and appear to be empty. (TIF) [file pgen.1008363.s007.tif]
